# Oridonin, a Promising *ent*-Kaurane Diterpenoid Lead Compound

**DOI:** 10.3390/ijms17091395

**Published:** 2016-08-24

**Authors:** Dahong Li, Tong Han, Jie Liao, Xu Hu, Shengtao Xu, Kangtao Tian, Xiaoke Gu, Keguang Cheng, Zhanlin Li, Huiming Hua, Jinyi Xu

**Affiliations:** 1 Key Laboratory of Structure-Based Drug Design & Discovery, Ministry of Education, and School of Traditional Chinese Materia Medica, Shenyang Pharmaceutical University, Shenyang 110016, China; lidahong0203@163.com (D.L.); hantong1221@163.com (T.H.); howlris@gmail.com (J.L.); huxu105@163.com (X.H.); kangtaotian@126.com (K.T.); lzl1030@hotmail.com (Z.L.); 2 Department of Medicinal Chemistry and State Key Laboratory of Natural Medicines, China Pharmaceutical University, Nanjing 210009, China; cpuxst@163.com; 3 Jiangsu Key Laboratory of New Drug Research and Clinical Pharmacy, Xuzhou Medical College, Xuzhou 221004, China; gu_xk@163.com; 4 State Key Laboratory for the Chemistry and Molecular Engineering of Medicinal Resources, and School of Chemistry and Pharmacy, Guangxi Normal University, Guilin 541004, China

**Keywords:** oridonin, *ent*-kaurane, medicinal chemistry, structural modification, biological activity

## Abstract

Oridonin belongs to *ent*-kaurane tetracyclic diterpenoid and was first isolated from *Isodon* species. It exhibits inhibitory activities against a variety of tumor cells, and pharmacological study shows that oridonin could inhibit cell proliferation, DNA, RNA and protein synthesis of cancer cells, induce apoptosis and exhibit an antimutagenic effect. In addition, the large amount of the commercially-available supply is also very important for the natural lead oridonin. Moreover, the good stability, suitable molecular weight and drug-like property guarantee its further generation of a natural-like compound library. Oridonin has become the hot molecule in recent years, and from the year 2010, more than 200 publications can be found. In this review, we summarize the synthetic medicinal chemistry work of oridonin from the first publication 40 years ago and share our research experience of oridonin for about 10 years, which may provide useful information to those who are interested in this research field.

## 1. Introduction

**Figure 1:**
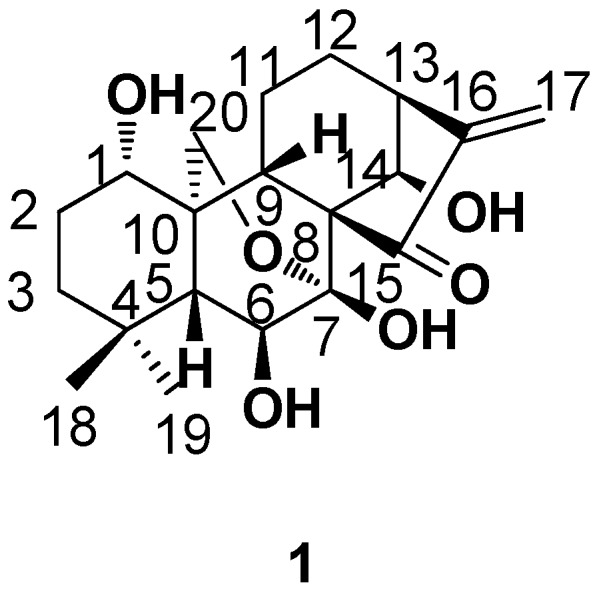
The structure and numbering of oridonin.

Oridonin (**1**, Figure 1) is an *ent*-kaurane diterpenoid isolated from *Isodon* of the Labiatae family, the structure and absolute configuration of which were first confirmed in the year 1970 [1]. Since then, hundreds of research articles, mainly in the antitumor field, have been published. Only from the year 2010, more than 200 papers can be found using Web of Science (searching oridonin as the topic), and obviously, it has become a molecule in focus from a natural source for the treatment of cancer. Oridonin is also a good lead in the field of medicinal chemistry: (a) it shows antitumor activities against many tumor-related cells [2,3] (for example, IC_50_ values were 21.48 μM against human esophageal squamous EC9706 cells [4], 5 μM against leukemia-derived Jurkat cells [5], 2.5 μM against human umbilical vascular endothelial cells [6], 15.6 μM against gastric cancer SGC-7901 cells [7], 19.32 μM against human pancreatic cancer BxPC-3 cells [8], 37.90 μM against human liver carcinoma HepG2 cells [9], 3.1 and 6.1 μM against uveal melanoma OCM-1 and MUM2B cells for a treatment of 24 h [10], 54.2 μM against human lung cancer A549 cells [11], 41.8 μM against leukemia HPB cells [12], 63.7 μM against human fibrosarcoma HT1080 cells [13], 15.18 μM against human epidermoid carcinoma A431 cells [14], 37.1 μM against human laryngeal cancer HEp-2 cells [15,16], 7.4 μM against melanoma K1735M2 cells [17], 3.88 μM against human colon tumor SW620 cells [18], 3.74 μM against bone marrow tumor K562 cells [18], 5.12 μM against breast tumor MCF7 cells [18], and so on) and relatively low toxicity (18.26 μM against human liver L-02 cells and 7.48 μM against human liver carcinoma Bel-7402 cells for a treatment of 72 h [19]); (b) the well-studied multitargeting properties of the antitumor activity [20,21] through specific chemical modifications enable the development of natural product (NP)-based novel drugs; (c) oridonin possessed good stability in stock solutions (1 mg/mL of oridonin and the internal standard were over 99% of the nominal concentrations compared with freshly prepared solutions after storage at −20 °C for 30 days), rat plasma (the starting concentrations were 12.3, 246 and 984 ng/mL; the measured concentrations were 12.0, 248 and 973 ng/mL after 24 h at room temperature and 12.1, 252 and 976 ng/mL at −20 °C for 30 days, respectively) and even after three freeze–thaw cycles (freezing at −20 °C and thawing at room temperature on three consecutive days) with concentrations of 11.8, 253 and 978 ng/mL [22,23]; (d) oridonin meets the criteria of Lipinski’s rule of five and is a lead-like, fragment-like and drug-like molecule [24,25]; the molecular weight (*M*w) of oridonin is 362.2, which makes it suitable for further optimization, because the *M*w value always increases during the process from lead to drug-like candidate [26]; (e) there are many functional groups (double bond, carbonyl group and hydroxyl groups in different chemical environments), which provide good synthetic accessibility to efficiently generate libraries of derivatives; and (f) last, but not least, for all natural products, it is commercially available for large-scale compound supply. Despite these promising profiles as an anticancer lead, there are also some shortcomings to be overcome during structural modification processes, such as relatively low aqueous solubility and bioavailability, moderate potency and undefined mechanisms of action. On the basis of the above, oridonin was widely used as a lead compound, especially to develop novel antitumor natural product-derived agents, by many medicinal chemistry researchers, including our research group [17,18,19,20,21,22,23,24,25,26,27,28,29,30,31,32,33,34,35,36,37,38,39,40,41]. With its molecular mechanisms being gradually clarified, more scientists will join this field to discover promising anti-neoplastic drug from oridonin, as well as tetracyclic diterpenoids. Although there are several reviews concerning the biological activities [20,21,42,43,44] and medicinal chemistry [45] (structural modification and biological evaluation) of oridonin, herein, we would like to review the synthetic work of the lead oridonin from the first publication 40 years ago and to share our research experience of oridonin for about 10 years, which not only provides the best convenience to whomever wants to join this research field, but also a good example for lead optimization research from natural sources.

## 2. Structure Modification on the Core Structure of Oridonin

**Figure 2:**
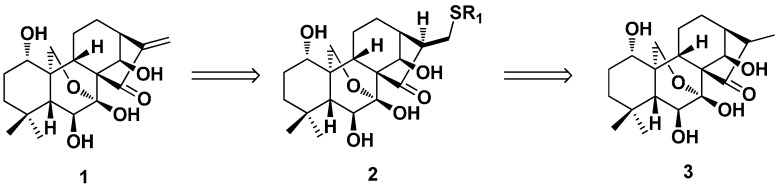
The biomimetic products (**2**) and dihydro-derivative (**3**) of oridonin.

In the year 1976, biomimetic reactions of oridonin and alkane thiols were done by Fujita et al. under mild conditions to give alkane thiol adducts (**2**) at C-17 [46]. The dihydro-derivative of oridonin (**3**) was obtained by the reduction reaction of **2** with Raney Ni (Figure 2). Compound **3** showed no antitumor activity against Ehrlich ascites carcinoma cells in mouse and antibacterial activity against 19 selected bacteria. Therefore, the α-methylene-cyclopentanone system in the structure of oridonin was first considered as an important active center.

**Figure 3:**
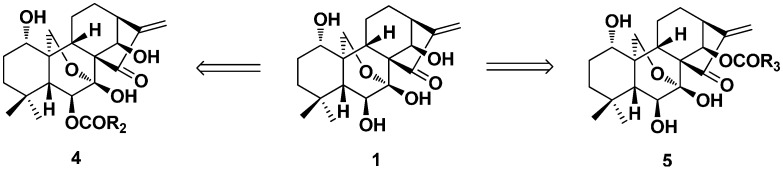
Selective acylated products of oridonin at 6-*O* (**4**) or 14-*O* (**5**).

Five years later (in 1981), Fujita et al. selectively synthesized the first series of derivatives of **1** by acylation at 6-*O* (**4**) or/and 14-*O* (**5**, Figure 3) [47]. The antitumor activity of synthetic derivatives against Ehrlich ascites carcinoma cells in mouse were evaluated, and the structure activity relationship (SAR) showed that the activity increased with the increase of the acyl carbon chain length. The importance of the hydrogen bond and the ester side chain for the antitumor activity was also demonstrated in the derivatives of oridonin; while the 1,14-diacetate derivative of oridonin could be obtained by the treatment of oridonin with acetic anhydride in pyridine [48].

**Figure 4:**
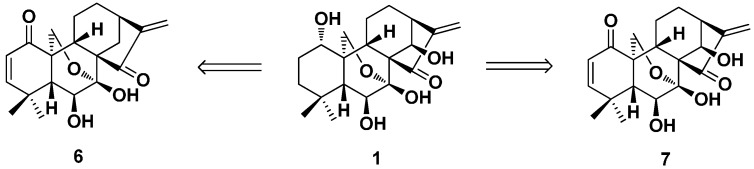
Synthesis of eriocalyxin B (**6**) and 14-hydroxyeriocalyxin B (**7**) from oridonin.

In 1990, eriocalyxin B (**6**) and its analogues were obtained by Zhou et al. from oridonin [49]. The overall yields of **6** and 14-hydroxyeriocalyxin B (**7**) were 11% and 57% in six and four steps, correspondingly (Figure 4).

**Figure 5:**
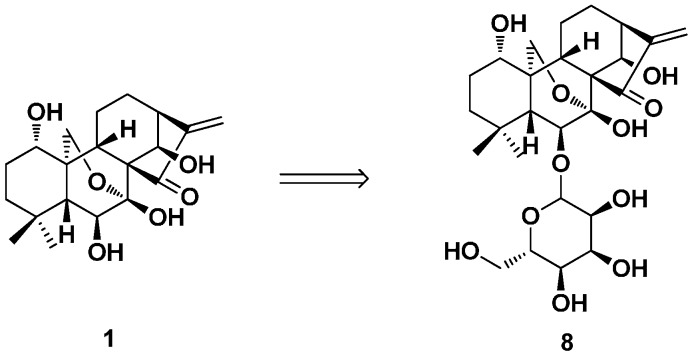
Synthesis of oridonin-6-*O*-α-d-glucopyranoside (**8**).

Oridonin-6-*O*-α-d-glucopyranoside (**8**) was synthesized by Liu’s group from **1** by five steps in a total yield of 23% (Figure 5). This method could improve the water solubility of oridonin derivatives [50].

**Figure 6:**
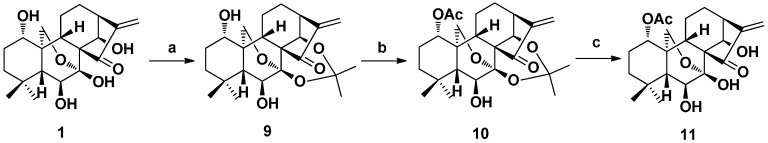
Synthesis routine of lasiokaurin (**11**). (**a**) 2,2-Dimethoxypropane, acetone, TsOH, 56 °C; (**b**) Ac_2_O, TEA (triethylamine), DMAP (4-dimethylaminopyridine), rt (room temperature); (**c**) 10% HCl, THF (tetrahydrofuran), rt.

1-OAc oridonin was also a natural product, which was called lasiokaurin (**11**). In 2006, Liu’s group synthesized **11** from **1** in a 69% overall yield via three steps by selective acetonide protection (**9**), acetylation (**10**) and deprotection (Figure 6). The antiprotozoan activity of lasiokaurin and oridonin was tested, and the median lethal concentration was 25 and 50 μM, respectively, which indicated that 1-hydroxyl had some benefits on the antiprotozoan activity [51]. These reactions were used extensively in the further modification of oridonin.

**Figure 7:**
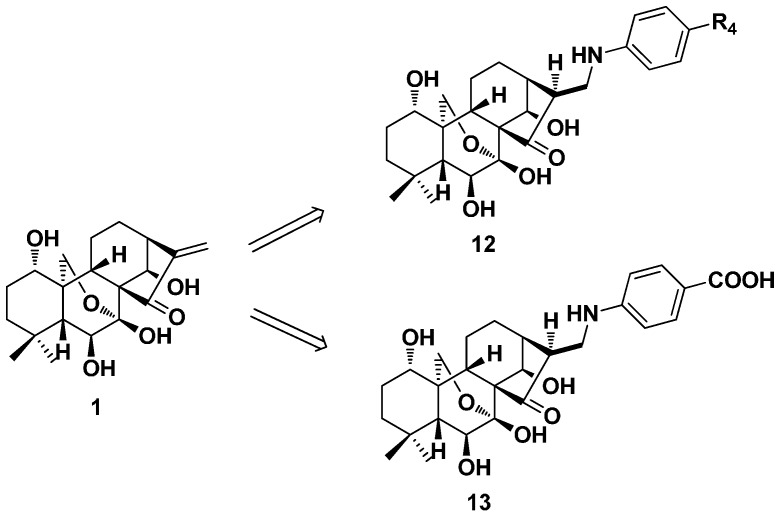
Aromatic amine derivatives (**12** and **13**) of oridonin.

In the next year, a series of aromatic amine derivatives (**12**, Figure 7) of oridonin was designed and obtained by Liu’s group [52]. The antiproliferative activity against oral epithelial KB cells was evaluated and all of the derivatives showed cytotoxicity to some extent, which was similar to or stronger than oridonin. When R_4_ was carboxyl, Compound **13** exhibited the strongest activity with an inhibition rate of 38.0% at a concentration of 0.8 mg/mL.

**Figure 8:**
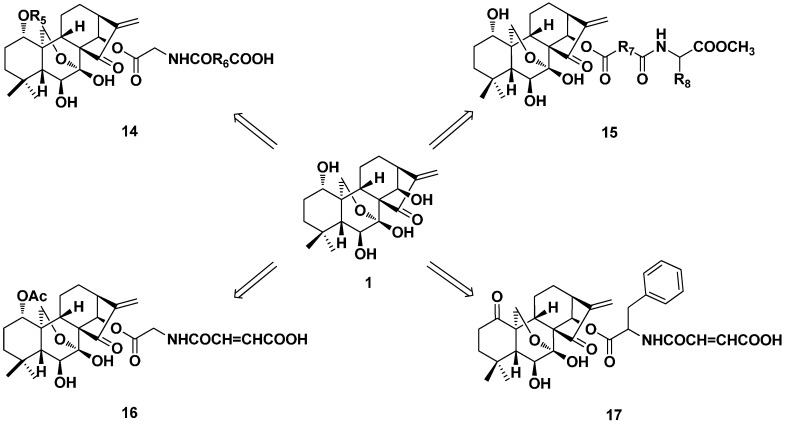
Synthesis of 1-*O* and 14-*O*-derivatives (**14**–**17**) of oridonin.

In the year 2008, our research group published our first work in the field of the structural modification of oridonin [41]. Some 1-*O* and 14-*O*-derivatives of oridonin were synthesized (Figure 8) and biologically evaluated against six cancer cell lines (SW-480, BGC-7901, HL-60, A549, Bel-7402 and B16). All of the derivatives exhibited stronger cytotoxicity than oridonin in vitro, and three of them were further evaluated in vivo. Derivatives **16** and **17** showed the most potent antiproliferative activity against HL-60 and Bel-7402 cell lines with the IC_50_ values of 0.84 and 1.00 μM, respectively. The preliminary SAR suggested that the introduction of both terminal carboxylic acid and the ester side chain of the lipophilicity moiety to the 14-*O* position of oridonin appeared to increase the antiproliferative activity. The cytotoxicity of the derivatives with the substituent of acetyl at the 1-*O* position was better than those with the propylsulfonyl group and the 1-hydroxyl oxidated derivatives. Compounds **16** and **17** also had stronger anti-tumor activity in mice bearing H22 liver tumor than oridonin (45.9%) with the inhibition ratios of 69.9% and 61.2%, correspondingly.

**Figure 9:**
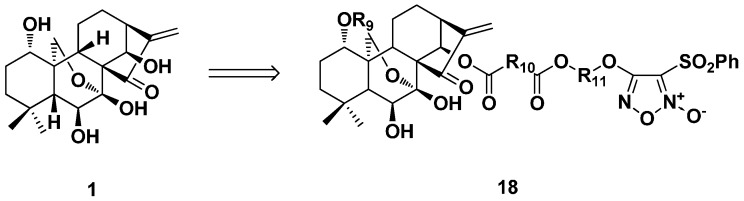
Synthesis of furoxan/oridonin NO-releasing hybrids (**18**).

Nitric oxide (NO) is a key mediator involved in many physiological and pathological processes. Li et al. synthesized several series of furoxan/oridonin hybrids (Figure 9) and evaluated the antiproliferative activity against four cancer cell lines (Bel-7402, CaEs-17, MGC-803, and K562) [38]. All of the target compounds released high levels of NO (more than 15 μM) at the time point of 60 min in vitro and exhibited stronger antiproliferative activity than the parent oridonin. Higher levels of NO-releasing capacity could be beneficial to cytotoxicity. In each series of the target synthetic hybrids, the derivative (**18**) with R_10_ of *o*-C_6_H_4_ and R_11_ of (CH_2_)_3_ exhibited the strongest antiproliferative activity among the designed hybrids with IC_50_ values in (sub)micromolar ranges. The linkages between the NO donor and drug molecule always affect the NO-releasing ability and biological activity.

**Figure 10:**
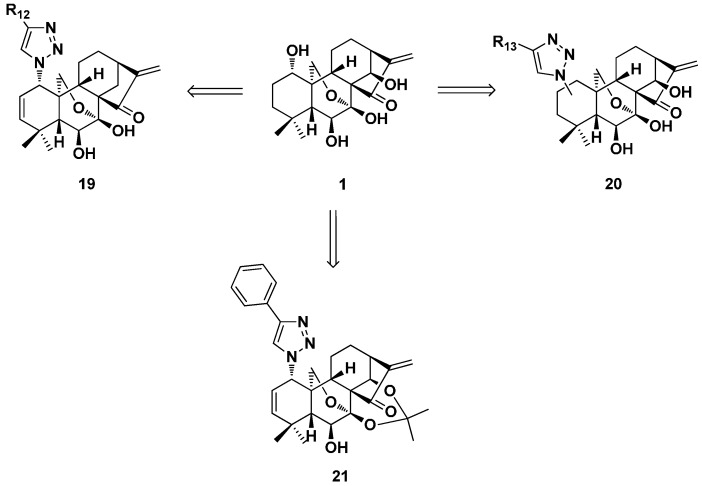
Installation of azides and 1,2,3-triazole at the C-1, -2, or -3 position derivatives (**19**–**21**) of oridonin.

In 2013, Zhou’s group developed an efficient and concise synthetic approach to install the azide functional group at the C-1, C-2 or C-3 positions of the A-ring of oridonin rapidly and diversely with highly-controlled regio- and stereo-selectivity (Figure 10) [53]. These azides were further functionalized through click chemistry to yield triazole derivatives. The antiproliferative activity of representative 1,2,3-triazolesubstituted derivatives (**19** and **20**) against breast cancer cell lines MCF-7 and MDA-MB-231 was tested. These derivatives with 1,2,3-triazole installed in the A-ring exhibited significantly improved antiproliferative activity compared to oridonin. Among them, Compound **21** showed the strongest potency with IC_50_ of 0.38 and 0.48 μM, respectively. This work provided access to an expanded potential anticancer natural scaffold-based compound library from the lead oridonin.

**Figure 11:**
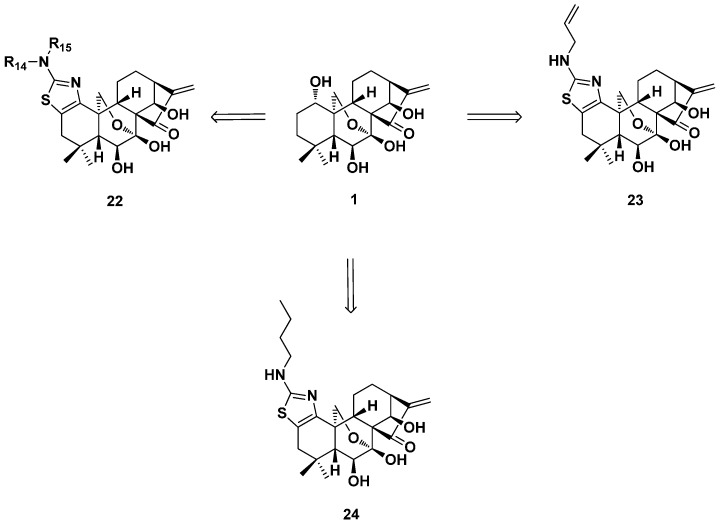
The thiazole-fused A-ring modified oridonin derivatives (**22**–**24**).

In the same year, Zhou’s group synthesized a series of oridonin derivatives (**22**) with thiazole fused in the A-ring through a protecting group-free synthetic strategy (Figure 11) [54]. Most of the derivatives exhibited potent antiproliferative effects against selected pancreatic, breast and prostate cancer cells with low (sub)micromolar IC_50_ values and enhanced aqueous solubility. These molecules not only showed enhanced growth inhibitory effects against MCF-7 cells, but also on the other oridonin insensitive cancer cells, including the highly invasive triple-negative MDA-MB-231 cell line with low IC_50_ values. Particularly, the most potent derivative (**23**) with an *N*-allyl substituted thiazole moiety exhibited IC_50_ values of 0.2 μM against both MDA-MB-231 and MCF-7 cells, which are approximately 147-fold and 33-fold more potent than oridonin, respectively. It was also found to induce the apoptosis of MCF-7/ADR and MDA-MB-231 cells in a concentration-dependent manner through similar multiple pathways as oridonin. The above derivative significantly suppressed MDA-MB-231 xenograft tumor growth at 5 mg/kg and was more efficacious than oridonin. Furthermore, one analogue (**24**, R_14_ = H, R_15_ = *n*-butane), which significantly inhibited HCC1806 and HCC1937 triple-negative breast cancer cells’ proliferation, was selected for an intensive mechanism study. The results showed that it could induce apoptosis and cell cycle arrest at the G_2_/M phase of HCC1806 and HCC1937 cells. The inhibitory potency would be caused by the expression of death receptor 5 (DR5), p21 and pERK and downregulations of cyclin D1, XIAP, FLIPL, pSTAT3 and pAKT. Besides, the suppression of HCC1806 xenograft tumor growth at 5 mg/kg in nude mice without the loss of body weight also guaranteed its further development as a drug candidate [55].

**Figure 12:**
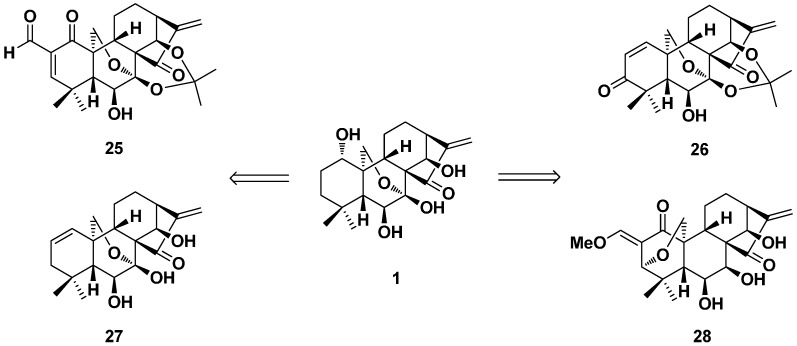
The dienone derivatives (**25**–**28**) of oridonin with the α,β-unsaturated ketone system in the A-ring.

In late 2013, a series of dienone derivatives of oridonin with an additional α,β-unsaturated ketone system installed in the A-ring was synthesized by Zhou’s group (Figure 12) [56]. Regioselective enone construction strategies were established. These derivatives significantly induced apoptosis and exhibited superior antitumor effects to oridonin against drug-resistant and aggressive breast cancer cells in vitro and in vivo (**26** suppressed MDA-MB-231 xenograft tumor growth at 5.0 mg/kg) and also exhibited low toxicity to normal human mammary epithelial cells. The preliminary mechanism studies revealed that selected dienone analogues (**25**, **26**) were found to induce the apoptosis of MDA-MB-231 cells in a concentration-dependent manner through regulation of a series of apoptotic-related proteins.

It was also reported that oridonin [57,58,59] and its derivatives [60,61] exhibited anti-inflammatory activity, such as the ability to treat hepatic fibrosis. The antifibrogenic effects of Compounds **27** [60] and **25** [61] were investigated on the activated rat HSC-T6 and human LX-2 stellate cell lines. These two derivatives could inhibit the proliferation of HSC-T6 and LX-2 cells in a dose- and time-dependent manner. However, for the human hepatocyte cell line C3A, no significant antiproliferative effects were observed. These two derivatives also induced apoptosis and cycle arrest at the S phase in the LX-2 cell line. The apoptosis-related property of Compound **27** was associated with the activation of p53, p21 and cleaved caspase-3, while Compound **25** could activate cleaved PARP, p21 and p53 and decrease cyclin-B1. Both of them could markedly downregulate major ECM proteins type I collagen and fibronectin and the myofibroblast marker protein *α*-smooth muscle actin in a time- and dose-dependent fashion and blocked transforming growth factor-*β*-induced type I collagen and fibronectin production. Oridonin analogues **25** and **27** would have great potential to act as promising antifibrogenic agents to treat hepatic fibrosis.

**Figure 13:**
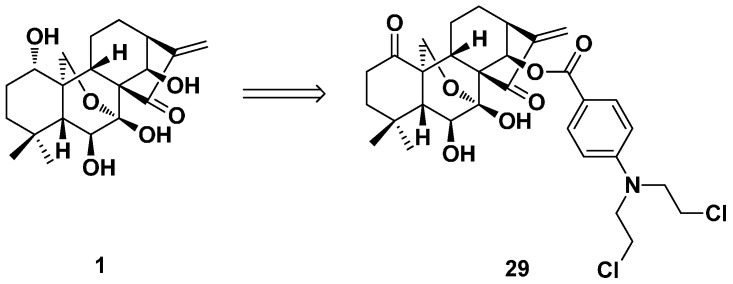
The hybrid of oridonin and nitrogen mustard (**29**).

In 2014, a series of oridonin and nitrogen mustard hybrids were designed and synthesized by Xu et al. to find more efficacious and less toxic antitumor agents (Figure 13) [30]. The antiproliferative activity of the hybrids was more potent than oridonin and the clinically used nitrogen mustards against four selected human cancer cell lines (Bel-7402, MCF-7, K562 and MGC-803). Some representative derivatives exhibited antiproliferative activities against the multidrug-resistant cell lines (NCI-H460/MX20 and SW620/AD300). The most effective compound (**29**) of this series showed strong inhibitory activity with an IC_50_ value (0.67 μM) 21-fold lower than that of oridonin (14.60 μM) against MCF-7 cells and also exhibited selective cytotoxicity toward different cancer cells. It was demonstrated to affect cell cycle progression and significantly induce apoptosis in human hepatoma Bel-7402 cells.

**Figure 14:**
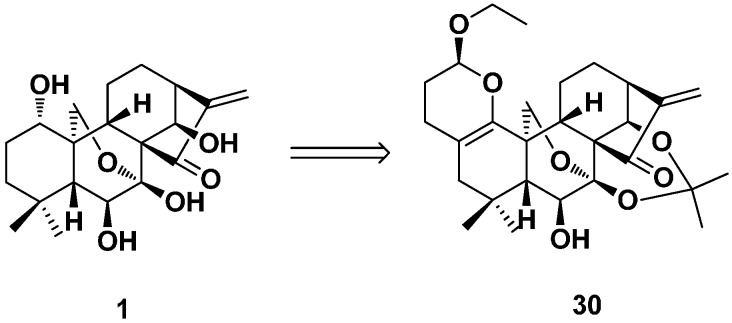
The dihydropyran-fused oridonin **30**.

In the same year, a series of dihydropyran-fused oridonin derivatives was designed and synthesized as potential anticancer agents by Zhou’s group (Figure 14) [62]. 3,4-dihydro-2*H*-pyran moiety was introduced into the A-ring of oridonin by an optimized IED HDA (inverse electron demand hetero-Diels–Alder) reaction in a mild and concise approach. The inhibitory effects of these derivatives were evaluated against MCF-7, MDAMB-231, MDA-MB-468 and MCF-7/ADR cell lines. Among them, Compound **30** was the most potent one, which showed submicromolar IC_50_ values in MCF-7 (IC_50_ = 0.44 μM), MDA-MB-231 (IC_50_ = 0.54 μM) and MDA-MB-468 (IC_50_ = 0.52 μM) cells and an improved capability to overcome chemoresistance against the MCF-7/ADR cell line (IC_50_ = 1.6 μM).

**Figure 15:**
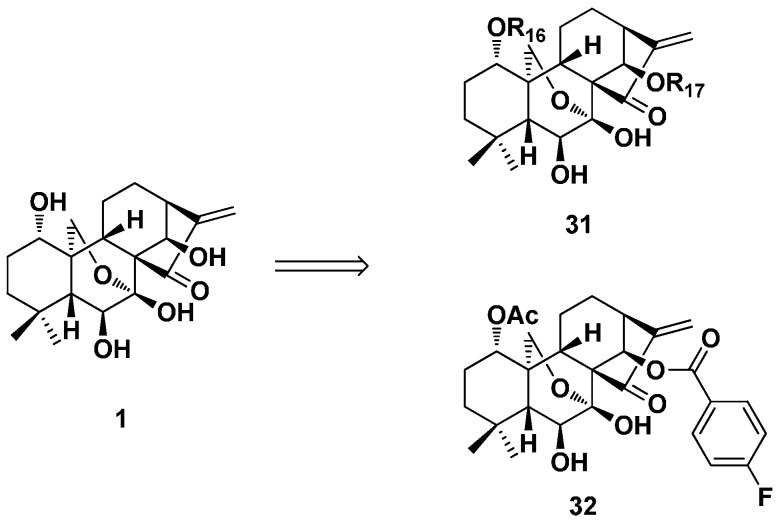
The 1- or/and 14-position modified oridonin derivatives (**31** and **32**) with antibacterial activity.

From 2014–2016, the antibacterial activity of 1- or/and 14-position modified oridonin derivatives (**31**) was evaluated (Figure 15) [29,31,32]. Some derivatives were screened against *Mycobacterium marinum*, *Mycobacterium smegmatis* and *Mycobacterium phlei*. Among them, the compounds containing the *trans*-cinnamic acid moiety showed the most potent inhibitory activity against *M. phlei* with MICs of 0.5 μg/mL, and the SARs were analyzed. Five compounds were tested against *Mycobacterium tuberculosis* H37Rv based on the preliminary screening results. Among them, Compound **32** showed an IC_50_ value of 17.1 μg/mL. The antibacterial activity of some derivatives against *Escherichia coli*, *Staphylococcus aureus*, *Bacillus subtilis* and *Monilia albicans* was evaluated for the first time. Most of them showed good antibacterial activity against Gram-positive bacteria *B. subtilis* and *S. aureus*. Additionally, no obvious inhibitory activity was observed against Gram-negative bacterium *E. coli* and fungus *M. albicans* (MIC > 100 μg/mL).

**Figure 16:**
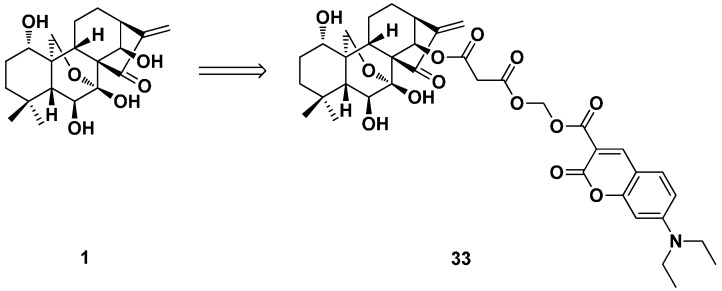
The oridonin fluorescent probe **33**.

Early in this year, a series of oridonin fluorescent probes linked with coumarin moieties were designed and synthesized to explore the anticancer mechanism by our research group (Figure 16) [27]. Most of the probe molecules displayed optimal antiproliferative activity and a fluorescent property. Fluorescence microscopy and confocal imaging studies were examined by using typical probes. The results indicated that oridonin fluorescent probe **33** could be rapidly taken up into tumor cells, and the mitochondrion was the main site where it accumulated. Moreover, we confirmed that the α,β-unsaturated ketone group is the active moiety, which is crucial to its cytotoxicity, localization and uptake. The studies on mitochondrial physiology suggested that the mitochondrion-related pathway was involved in oridonin-induced apoptosis, and cytochrome c played an important role. These results provide new insights into the cellar mechanism of oridonin and may be useful to study the insight of the anticancer action and the targets of other *ent*-kaurane diterpenoids.

## 3. Oridonin as the Lead to Synthesize *ent*-Kaurane Diterpenoid Derivatives of Other Types

*ent*-Kaurane diterpenoid derivatives with diverse unique chemical skeletons are generally quite complex, incorporating numbers of intricate ring systems and stereogenic centers, and exhibit promising biological activities [63,64,65,66,67,68,69]. The preparation of *ent*-kaurane diterpenoid libraries inevitably involved rather laborious and complex synthetic sequences, especially total synthesis [70,71,72]. Therefore, the therapeutic development of these compounds was significantly impeded by the problem of large-scale compound supply. It is found that oridonin is a good relevant commercially-available lead to semi-synthesize novel *ent*-kaurane diterpenoid derivatives with different scaffolds bypassing the de novo synthetic strategy.

### 3.1. 15,16-seco-ent-Kaurane Diterpenoid Derivatives

**Figure 17:**
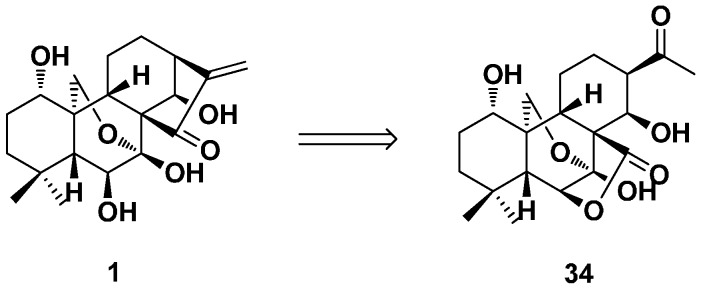
The synthesis of 15,16-*seco*-*ent*-kaurane rubescensin S (**34**) from oridonin.

In the year 2011, Zhang et al. synthesized rubescensin S (**34**) from oridonin by two steps and revised its stereochemistry (Figure 17) [73]. An effective two-step transformation from oridonin to the 15,16-*seco*-*ent*-kaurane skeleton (rubescensin S) was achieved. This key compound provided a good building block for further construction of a natural product-like 15,16-*seco*-*ent*-kaurane compound library. They also revised its structure of the 13*S* configuration instead of the reported 13*R*.

### 3.2. 6,7-seco-ent-Kaurane Diterpenoid Derivatives

**Figure 18:**
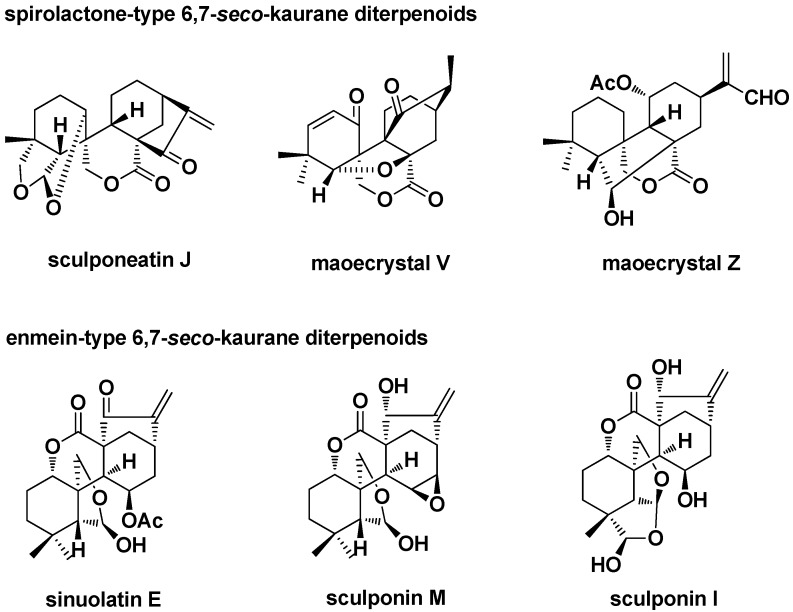
Natural spirolactone- and enmein-type 6,7-*seco*-kaurane diterpenoids.

6,7-*seco*-*ent*-Kaurane diterpenoid derivatives (including enmein- and spirolactone-type 6,7-*seco*-*ent*-kaurane diterpenoids) showed stronger cytotoxicity than oridonin. These compounds also have more complex structures (Figure 18) and are more difficult to isolate from natural sources [74,75,76,77,78,79]. We used oridonin as the starting material to semi-synthesize enmein- and spirolactone-type core structures by the ring-opening reaction between C-6 and C-7. A compound library containing more than 100 enmein- and spirolactone-type diterpenoid derivatives was built up mainly by our research group for further medicinal study.

#### 3.2.1. Enmein-Type 6,7-*seco*-*ent*-Kaurane Diterpenoid Derivatives

**Figure 19:**
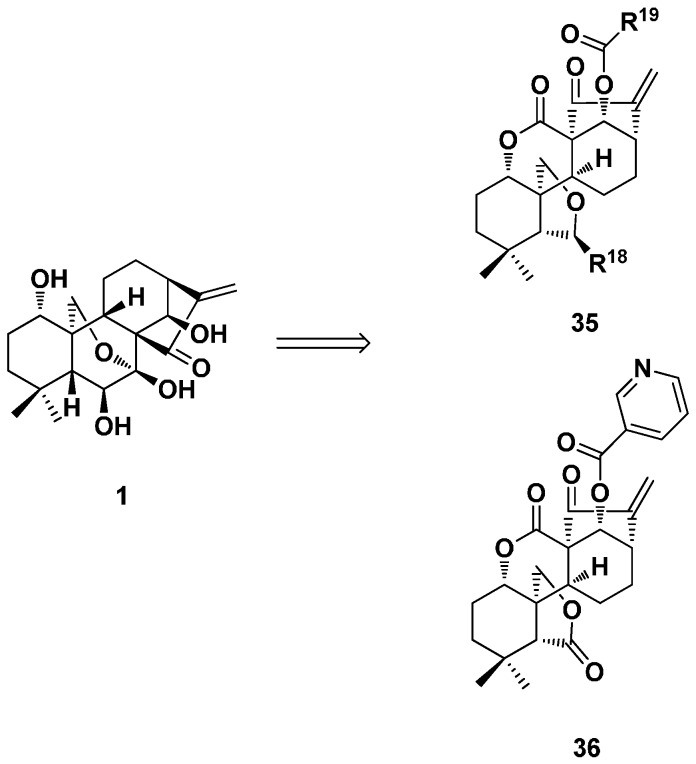
Enmein-type 6,7-*seco*-kaurane diterpenoid derivatives **35** and **36**.

Several series of ester derivatives (**35**) of enmein-type diterpenoid at 14-*O* were synthesized from commercially-available oridonin by efficient and practical synthetic methods (Figure 19) [33,36]. The antiproliferative activity was evaluated against a set of human cancer cell lines. Some derivatives showed even smaller IC_50_ values than positive control paclitaxel. The apoptotic properties of the selected Compound **36** (IC_50_ = 0.71 μM) in human hepatocarcinoma Bel-7402 cells were evaluated. It caused cell-cycle arrest at the G_2_/M phase and induced apoptosis. Moreover, Compound **36** exhibited potent antitumor activity in vivo in MGC-803 mice. These results warranted further preclinical investigations of these enmein-type diterpenoid derivatives as potential anticancer agents.

**Figure 20:**
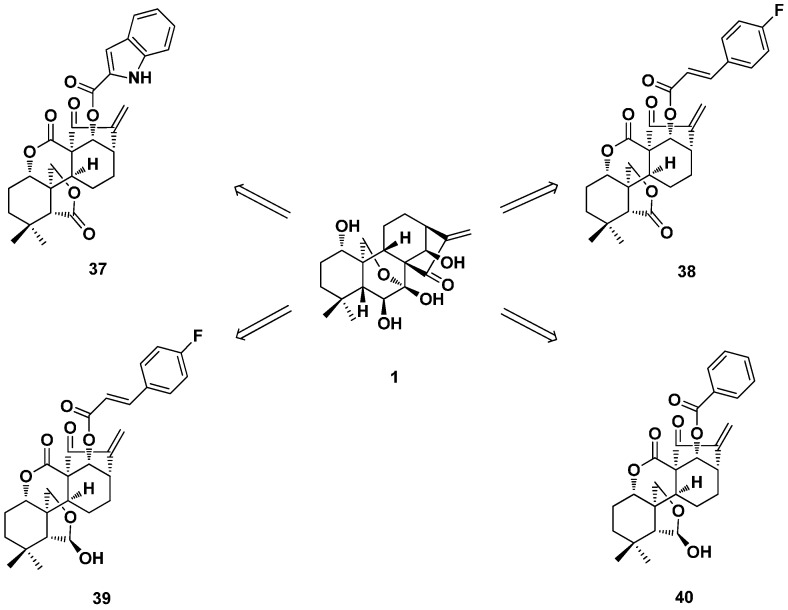
Antimycobacterial enmein-type 6,7-*seco*-kaurane diterpenoid derivatives **37**–**40**.

The antimycobacterial activity of some enmein-type 6,7-*seco*-kaurane diterpenoid derivatives (**35**) was also evaluated [31,32]. Most of the derivatives showed antimycobacterial activity against *M. phlei*, of which Compounds **37**–**39** (Figure 19) exhibited the strongest activity with MIC of 0.5 μg/mL and were 15-fold stronger than that of oridonin (8 μg/mL). The *trans*-cinnamic acid moiety benefitted the antimycobacterial activity. Compounds **38** and **40** (Figure 20) also showed moderate antitubercular activity against *M. tuberculosis* H_37_Rv with MICs of 28.8 and 24.0 μg/mL, respectively. These findings could provide new insights into the development of novel antitubercular agents from enmein-type 6,7-*seco*-kaurane diterpenoid derivatives.

**Figure 21:**
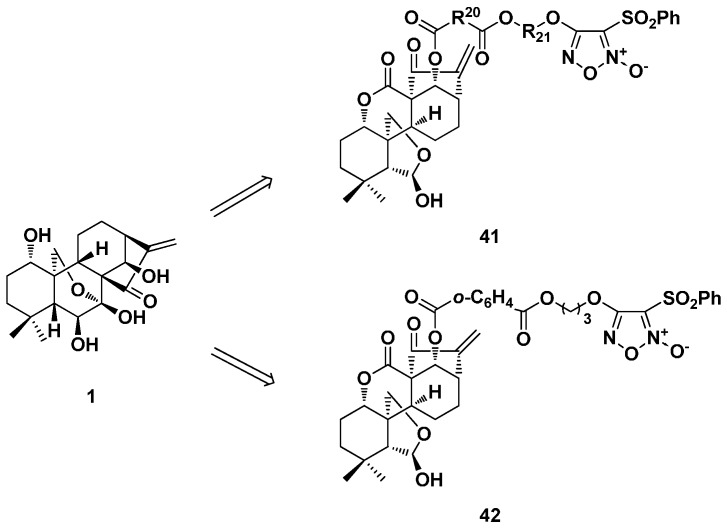
NO-releasing enmein-type 6,7-*seco*-kaurane diterpenoid derivatives **41** and **42**.

In this year, a series of NO-donating enmein-type diterpenoid derivatives (**41**) was designed and synthesized (Figure 21) [19]. The target derivatives showed potent antibacterial activity against Gram-positive bacteria *S. aureus* and *B. subtilis* with the most promising MICs of 4 and 2 μg/mL, respectively, while the MICs of oridonin were both 32 μg/mL. The antiproliferative activity against human tumor and human normal cells was also tested. Most of these NO-releasing molecules showed good cytotoxic selectivity and released high levels (above 20 μmol/L) of NO at the time point of 60 min. Compound **42** was the most promising one with IC_50_ values of 1.68, 1.11, 3.60 and 0.72 μM against K562, MGC-803, CaEs-17 and Bel-7402 cells and 18.80 μM against normal liver cell line L-02. The selectivity index (SI) of **42** between tumor and normal liver cells was about 26.1, while the SI of oridonin was only 2.4. Compound **42** also induced apoptosis by the mitochondria-related pathway and arrested Bel-7402 cell cycle at the S phase.

#### 3.2.2. Spirolactone-Type 6,7-*seco*-*ent*-Kaurane Diterpenoid Derivatives

**Figure 22:**
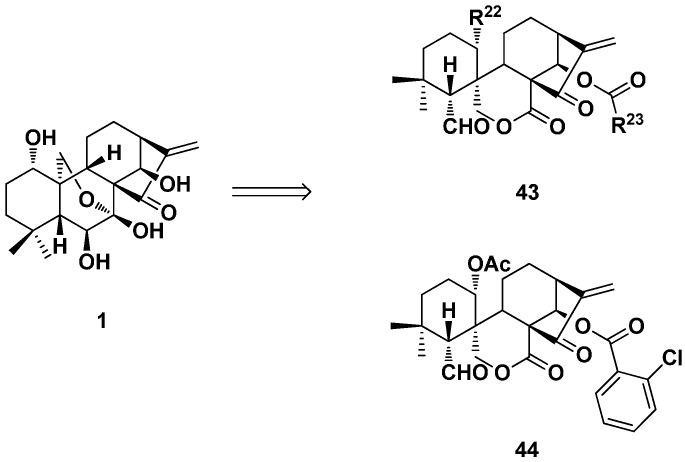
Spirolactone-type 6,7-*seco*-kaurane diterpenoid derivatives **43** and **44**.

The spirolactone-type 6,7-*seco*-*ent*-kaurane diterpenoid derivatives (**43**) were also obtained from commercial-available oridonin (Figure 22) [34,37]. These derivatives showed improved antiproliferative activity against a panel of human cancer cell lines, and some of them were more potent than the positive control Taxol. For example, the most potent Compound **44** with chloro substitution at the *ortho*-position of the benzene ring showed IC_50_ values of 0.39, 1.28, 0.60 and 1.39 μM against K562, MGC-803, CaEs-17 and Bel-7402 cells, which were 11.2-, 3.4-, 17.4- and 4.3-fold stronger than oridonin, correspondingly. The cellular mechanisms showed that Compound **44** could induce apoptosis at low micromolar concentrations in human hepatoma Bel-7402 cells.

### 3.3. ent-Kaurane Diterpenoid Dimers

**Figure 23:**
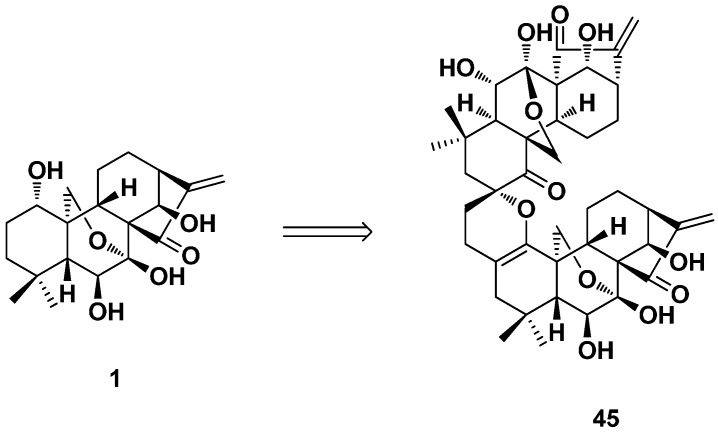
*ent*-Kaurane diterpenoid dimer **45**.

Many *ent*-kaurane diterpenoid dimers with diverse structures were isolated from natural sources [80,81,82]. It was found that oridonin could be a good lead to semi-synthesize *ent*-kaurane diterpenoid dimer derivatives to achieve the final drug candidate. A 3,4-dihydro-2*H*-pyran ring was firstly constructed in the A-ring of oridonin using an optimized IED HDA (inverse electron demand hetero-Diels–Alder) reaction. *ent*-Kaurane diterpenoid dimers were synthesized through a homo-HDA reaction by a self-dimerization of the exocyclic enone in the A-ring (Figure 23) [62]. The antiproliferative effects were evaluated against four breast cancer cell lines, MCF-7, MDA-MB-468, MDAMB-231 and MCF-7/ADR, by the MTT method, and the IC_50_ values were in the submicromolar range with a significantly improved capability to overcome chemoresistance.

## 4. Conclusions

In summary, there is an increasing number of studies advocating the medicinal chemistry field of the lead oridonin especially in the treatment of cancer. We propose *ent*-kaurane tetracyclic diterpenoids as natural multi-target agents represent promising therapeutic agents. However, further studies are required to elucidate the detailed molecular mechanisms of their actions. Structural modification should focus on the enhancement of potency and activity spectrum and, thereby, counter resistance mechanisms. Meanwhile, improving water solubility, the reduction of toxicity and increasing metabolic stability will continue to make significant contributions to the drug development of oridonin. Furthermore, the synthesis of some bioactive natural rare *ent*-kaurane diterpenoid analogues will be another key research direction for oridonin, which will provide structurally simpler compounds with retained bioactivities. It is also an economic strategy for the development of natural product-derived drugs.

## Abbreviations

*M*w	molecular weight
NP	natural product
SAR	structure activity relationship
DMAP	4-dimethylaminopyridine
NO	nitric oxide
rt	room temperature
THF	tetrahydrofuran
TEA	triethylamine
Ac	acetyl group
MTT	3-(4,5-dimethylthiazol-2-yl)-2,5-diphenyltetrazolium bromide
MIC	minimal inhibitory concentration
DR5	death receptor 5
IED	inverse electron demand
HDA	hetero-Diels–Alder
SI	selectivity index
